# Dexmedetomidine versus remifentanil for sedation during awake intubation using a Shikani optical stylet: a randomized, double-blinded, controlled trial

**DOI:** 10.1186/s12871-016-0219-9

**Published:** 2016-08-02

**Authors:** Ting Xu, Min Li, Cheng Ni, Xiang-yang Guo

**Affiliations:** Department of Anesthesiology, Peking University Third Hospital, Beijing, 100191 China

**Keywords:** Dexmedetomidine, Remifentanil, Shikani optical stylet, Awake intubation, Sedation

## Abstract

**Background:**

The purpose of this study was to compare the efficacy and safety of dexmedetomidine versus remifentanil for sedation during awake intubation using a Shikani optical stylet (SOS).

**Methods:**

Sixty-eight patients with cervical trauma or severe cervical spondylosis undergoing cervical spinal surgery were enrolled in this prospective study. They were randomly assigned to receive dexmedetomidine (Group D) or remifentanil (Group R). In Group D, the patients received an intravenous loading dose of dexmedetomidine 1 μg · kg^−1^ over 10 min followed by a continuous infusion of 0.7 μg · kg^−1^ · h^−1^. In Group R, a target-controlled infusion of remifentanil was administered to achieve a plasma concentration of 2.5 ng · ml^−1^, increased to 3 ng · ml^−1^ 10 min later. An endotracheal tube was inserted using a SOS under dexmedetomidine or remifentanil sedation after topical anesthesia to the airway. Midazolam was given as rescue sedation. We recorded the first attempt intubation success rate, the dose of midazolam, duration of intubation, Ramsay Sedation Scale (RSS) score, tracheal tube tolerance score, duration of drug infusion, adverse events and patient satisfaction score.

**Results:**

The RSS score was significantly higher in Group D than in Group R. First attempt success rate, rescue midazolam dose and the duration of intubation did not differ between the groups. Patients in Group R were significantly more tolerant of the tracheal tube. The incidence of hypoxia was significantly higher in Group R than Group D, but there was no significant difference in the incidence of other adverse events between the groups. The hemodynamic responses of the two groups were similar, but more patients in Group R were able to recall airway instrumentation.

**Conclusions:**

Both dexmedetomidine and remifentanil are effective sedatives for awake intubation using an SOS. Although the first attempt success rates were similar, patients sedated with remifentanil tolerated the tracheal tube better after intubation with moderately increased risk of desaturation.

**Trial registration:**

www.chictr.org.cn; ChiCTR-TRC-13003052 (February 4th, 2013).

## Background

The Shikani optical stylet™ (SOS; Clarus Medical, Minneapolis, MN, USA) is a rigid but malleable stylet with fiberoptic rods and a lens. The SOS is an effective means of managing patients with a difficult airway [1–6]. Compared with a Macintosh laryngoscope, use of a SOS reportedly reduces cervical spine movement during intubation [7], suggesting that it might reduce the risk of spinal cord injury during instrumentation of the airway in patients with a potential or documented cervical spine injury.

The SOS is most often used to intubate patients under general anesthesia. Awake intubation using the SOS in a patient with a difficult airway has been reported in two cases [3, 8]. The best means of providing sedation in awake SOS intubation has not yet been established. Sedation is one of the key elements for successful awake intubation. Intravenous midazolam, propofol and remifentanil are commonly used as sedatives for awake fiberoptic endoscope intubation (AFOI), and there have been recent reports of the safe and effective use of dexmedetomidine [9–14]. Dexmedetomidine is a highly selective α_2_ adrenoceptor agonist that acts as a sedative, analgesic and a moderate antisialagogue without respiratory depression, suggesting that it could be a suitable drug for facilitating awake intubation. Previous studies have demonstrated that dexmedetomidine is superior to midazolam [13], fentanyl [15], propofol [16] and sufentanil [17] in AFOI. Two studies compared dexmedetomidine with remifentanil for AFOI, but their results were contradictory [9, 10].

The sedation regimen for AFOI might not be suitable for awake intubation when a SOS is used instead of a fiberoptic bronchoscope (FOB), as the two devices have different characteristics and are manipulated differently. This study was designed to compare the efficacy and safety of dexmedetomidine with that of remifentanil during awake intubation with a SOS.

## Methods

After institutional ethics committee approval (IRB00006761-2012045, Medical Ethics Committee of Peking University Third Hospital), informed written consent was obtained from all patients. We enrolled patients between December 2013 and December 2014. Inclusion criteria were: age 18–70 years; American Society of Anesthesiologists (ASA) physical status score I–III; requirement for preoperative neck immobilization with a hard plastic collar; planned urgent or elective cervical spine surgery for cervical trauma or severe cervical spondylosis. Exclusion criteria were: pregnancy; use of an α_2_ adrenoreceptor agonist or antagonist within the previous 14 days; known or admitted alcohol or drug misuse; uncontrolled seizure disorder; history of unstable angina or myocardial infarction; resting heart rate (HR) <50 min^−1^; and complete heart block.

Patients were assigned by a computer-generated randomization schedule to receive sedation with dexmedetomidine (Group D) or remifentanil (Group R). A research nurse generated the allocation sequence, enrolled participants and assigned them to their groups. While one anesthesiologist prepared and infused the study drug, another anesthesiologist experienced in the use of the SOS was in charge of airway anesthesia and intubation. Another research nurse assessed the patients, recorded intubation time and followed up the patients postoperatively. The participants, the intubating anesthesiologist and the nurse who was responsible for assessment and follow up were blinded to the group allocation.

All patients received a bolus of intravenous scopolamine 0.3 mg as premedication and oxygen by nasal cannula (3 L · min^−1^). Vital signs, including systolic blood pressure (SBP), diastolic blood pressure (DBP), HR and pulse oxygen saturation (SpO_2_) were recorded at baseline and every 2 min until the completion of intubation. The time required for intubation (from the first insertion of the SOS to confirming intubation with capnography) and the number of attempts was also recorded.

All patients received the study drug via an Alaris PK Syringe Pump (Care Fusion, Becton Dickinson, Franklin Lakes, NJ). The study drug was diluted to 50 mL with a 0.9 % NaCl solution, and the infusion was started 10 min before airway anesthesia and continued throughout airway management and intubation. Group D received a loading dose of 1.0 μg · kg^−1^ dexmedetomidine over 10 min followed by a continuous infusion of 0.7 μg · kg^−1^ · h^−1^ [12]. Group R received a target-controlled infusion of remifentanil using the Minto three compartment model. The initial target was set at 2.5 ng · ml^−1^ and increased to 3 ng · ml^−1^ 10 min later. In both groups, the drug infusion was continued until confirmation of successful intubation.

During application of topical airway anesthesia and intubation, the cervical collar was not released. Airway anesthesia began 10 min after the start of sedative drug infusion. Lidocaine 200 mg was administered through a laryngotracheal mucosa atomization device (LMA MADgic, Teleflex Medical, Athlone, Republic of Ireland) to the mouth, larynx and glottis.

The application of topical anesthesia to the upper airway took no less than 10 min. The patient’s sedation level was assessed using the Ramsay Sedation Scale (RSS) at baseline, 10 min after the drug infusion had started, and every 3 min during airway anesthesia. Any patient with an RSS <2 was given a rescue bolus of intravenous midazolam 0.5 mg until an RSS of 2 was achieved [12].

A SOS preloaded with an endotracheal tube (ETT) was inserted over the tongue. The supine patient was asked to take deep breaths. The epiglottis and the glottic opening were identified via the eyepiece. Once the vocal cords were visualized, the tip of the ETT was advanced during inspiration. After the tip of ETT had entered the trachea, the SOS was withdrawn. Intubation score was assessed using a 5-point scale during SOS endoscopy and intubation (1, no movement; 2, grimacing; 3, mild cough; 4, major limb movement; 5, prolonged coughing) [13]. If the intubation score was >2 during endoscopy, the SOS was withdrawn and 3 mL 2 % lidocaine was sprayed on to the glottis via the LMA MADgic. The patient’s sedation level was reassessed and rescue midazolam 0.5 mg was given repeatedly in 1-min intervals until RSS ≥2.

The SOS was withdrawn if the patient’s SpO_2_ was ≤92 % during endoscopy. Oxygen was given via facemask (5 L · min^−1^) and the patient was instructed to take deep breaths. When SpO_2_ recovered to ≥95 %, another intubation attempt was made.

Immediately after intubation, end tidal CO_2_ concentration (first breath) was recorded. Tolerance of the ETT was assessed using a 3-point scale (1, well tolerated and cooperative; 2, mild coughing and/or grimacing but still cooperative; 3, severe coughing and/or agitated and not cooperative) [15]. General anesthesia was induced immediately after assessment of ETT tolerance. Infusion of the study drug was discontinued upon completion of induction of general anesthesia.

At the 24-h postoperative follow-up visit, patients were interviewed to assess their recall of pre-anesthesia events, administration of topical anesthesia, endoscopy and intubation, and whether there had been complications (for example, injury to the teeth, lip or oral mucosa, sore throat or hoarseness). Patient satisfaction with the whole procedure was assessed on an 11-point numeric rating scale (0, completely dissatisfied; 10, completely satisfied).

The primary efficacy endpoint of this study was the proportion of patients intubated successfully at the first attempt. Based on the findings of a previous study, in which the first attempt success rates using dexmedetomidine versus remifentanil in AFOI were 38 and 76 % respectively [10], we calculated that a sample size of 64 patients would be sufficient to detect a difference between the treatment groups with a power of 0.8 and a significance level of 0.05. Considering possible 5 % dropout, the sample size was set at 68.

We used SPSS 13.0 software (SPSS, Chicago, IL) for statistical analyses. Continuous variables are expressed as mean ± standard deviation, and were compared within groups using the paired *t*-test and between groups using the independent *t*-test. The chi-squared test or Fisher’s exact test were used to compare categorical data between the groups. Intubation conditions and tolerance score were analyzed using the independent samples Mann–Whitney *U* test. Blood pressure and HR at different time points were compared using two-way repeated-measures analysis of variance. A *P* value <0.05 was regarded as statistically significant.

## Results

**Fig. 1:**
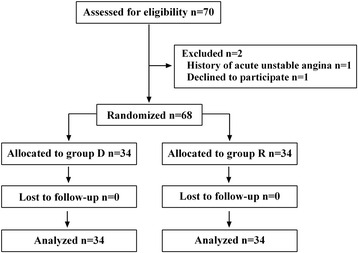
Study flow diagram. Group D, group Dexmedetomidine; Group R, group remifentanil

A total of 70 patients were assessed for eligibility, and 68 patients were enrolled. The enrolled patients were randomized and all of them completed this study (Fig. 1). Patients’ demographic and clinical characteristics did not differ between the groups (Table 1).

**Table 1:** Demographic and clinical characteristics of study participants

Characteristic	Group D (*n* = 34)	Group R (*n* = 34)	*P* value
Age (years)	52.9 ± 10.0	50.9 ± 9.4	0.412
Sex (male/female)	18(53)/16(47)	21(62)/13(38)	0.462
Cervical trauma/cervical spondylosis	15(44)/19(56)	18(53)/16(47)	0.467
ASA status (I, II or III)	10(29)/22(65)/2(6)	14(41)/17(50)/3(9)	0.958
Mallampatti grade (1, 2, 3 or 4)	9(26)/16(47)/8(24)/1(3)	8(24)/18(53)/6(18)/2(6)	0.947
BMI (kg · m^−2^)	25.5 ± 3.9	25.3 ± 3.3	0.864
Mouth opening (cm)	4.7 ± 0.7	4.6 ± 0.9	0.928

Data are presented as mean ± standard deviation or number (proportion, %)

*Abbreviations*: *BMI* body mass index, *ASA* American Society of Anesthesiologists

The baseline RSS of the two groups were similar (Table 2), but RSS was significantly higher in Group D than in Group R 10 min after drug infusion (Table 2, *P* = 0.001).

**Table 2:** Airway management characteristics

	Group D (*n* = 34)	Group R (*n* = 34)	*P* value
RSS 10 min after start of drug infusion	3 (2,4)	2 (2,4)	0.001
Intubation score at first attempt	2 (1,4)	2 (1,3)	0.119
First intubation attempt success rate (%)	79.4	85.3	0.525
Intubation attempts (1/2/3/4)	27(79)/0/5(15)/2(6)	29(85)/1(3)/4(12)/0	0.442
Cumulative dose of midazolam (mg)	1.1 ± 0.2	0.9 ± 0.2	0.109
Duration of drug infusion (min)	26.0 ± 4.5	24.8 ± 4.0	0.247
Duration of intubation(s)	107 ± 153	76 ± 106	0.342
Tolerance of endotracheal tube (1/2/3)	9(26)/24(71)/1(3)	22(65)/12 (35)/0	0.001
First P_ET_CO_2_ (cmH_2_O)	36.3 ± 4.0	36.4 ± 3.5	0.897
Incidence of recall			
Pre-anesthesia events	34 (100)	34 (100)	1.000
Topical anesthesia	29 (85)	34 (100)	0.020
Endoscopy	20 (59)	33 (97)	0.000
Intubation	8 (34)	26 (64)	0.000
Patient satisfaction score	7.9 ± 0.8	7.9 ± 1.0	0.792

Data are presented as median (minimum, maximum), mean ± standard deviation or number (proportion, %)

*Abbreviations*: *RSS* Ramsey sedation score, *P*_*ET*_ *CO*_*2*_ % end tidal carbon dioxide

All patients were successfully intubated with the SOS. The first intubation attempt success rates and the need for rescue midazolam were similar between the groups (Table 2). In first-time SOS insertions, the intubation scores of the two groups were broadly comparable. After intubation, mild coughing was observed in 12 patients in Group R, compared with 24 patients in Group D (and one case of severe coughing), resulting in significantly different tube tolerance scores (*P* = 0.001). The duration of drug infusion was 26.0 ± 4.5 versus 24.8 ± 4.0 min in Groups D and R, respectively (*P* = 0.247).

Nine patients in Group R and two in Group D developed hypoxia during intubation. The incidence of hypoxia (defined as SpO_2_ ≤ 90 % or a decrease of 10 % below baseline saturation, with the number of desaturation episodes measured on a per patient basis) in Group R was significantly higher than Group D (26 % versus 6 %, *P* = 0.021). The lowest SpO_2_, observed in a patient in Group R, was 85 %. All patients who developed hypoxia recovered to an SpO_2_ ≥ 95 % within 2 min after administration of supplementary oxygen by face mask. The hemodynamic changes observed in both groups were similar (Table 3). There were no significant differences in the occurrence of hemodynamic adverse events between the groups from the beginning of drug infusion until 10 min after intubation (Table 4). Significantly more patients in Group R recalled airway management than Group D (Table 2). There were no significant differences in patient satisfaction (Table 2) or intubation complications between the two groups (Table 4).

**Table 3:** Hemodynamic changes during intubation (*n* = 34)

Index	Group	T1	T2	T3	T4	*P*_(between groups)_
SBP (mmHg)	D	130 ± 18	120 ± 18	127 ± 30	148 ± 16	0.630
	R	131 ± 14	122 ± 14	125 ± 15	137 ± 13	
DBP (mmHg)	D	77 ± 13	72 ± 11	75 ± 11	87 ± 12	0.761
	R	79 ± 10	73 ± 13	74 ± 12	82 ± 13	
HR (min^−1^)	D	79 ± 16	72 ± 16	78 ± 15	86 ± 14	0.682
	R	76 ± 12	72 ± 12	77 ± 11	85 ± 12	

Data are presented as mean ± standard deviation. T1, baseline; T2, 10 min after study drug infusion; T3, pre-intubation; T4, 1 min after intubation

*Abbreviations*: *SBP* systolic blood pressure, *DBP* diastolic blood pressure, *HR* heart rate

**Table 4:** Adverse events during airway management

Adverse event	D	R	*P*-value
Hypotension	3 (8.8)	1 (2.9)	0.303
Hypertension	3 (8.8)	6 (17.6)	0.283
Tachycardia	7 (20.6)	9 (26.5)	0.567
Bradycardia	2 (5.9)	0 (0)	0.151
Hypoxia	2 (5.9)	9 (26.5)	0.021
Loosening of teeth	0 (0)	0 (0)	1.000
Injury to lip or oral mucosa	0 (0)	0 (0)	1.000
Postoperative sore throat	16 (47.1)	18 (52.9)	0.628
Hoarseness	2 (5.9)	2 (5.9)	1.000

Data are given as number (proportion, %). Baseline values of systolic blood pressure (SBP), diastolic blood pressure (DBP) and peripheral oxygen saturation (SpO_2_) were used to define adverse events. Hypotension was defined as SBP <80 mmHg, DBP <50 mmHg or SBP decreased ≤30 % below baseline values. Hypertension was defined as SBP >180 mmHg, DBP >100 mmHg or an SBP increased ≥30 % higher than baseline values. Bradycardia was defined as HR <45 min^−1^ or a decrease to ≤30 % below baseline. Tachycardia was defined as HR >120 min^−1^ or an increased ≥30 % higher than baseline values. Hypoxia was defined as SpO_2_ ≤ 90 % or a decrease by ≥10 % of the baseline saturation

## Discussion

We found that remifentanil and dexmedetomidine are both suitable for use as sedatives for awake SOS intubation. Although the first attempt success rates were similar between the groups, patients sedated with remifentanil tolerated the tracheal tube better after intubation, but at the expense of a greater risk of mild desaturation.

We selected the dose of the sedative according to previous studies of AFOI. The loading dose of 1 μg · kg^−1^ over 10 min followed by a continuous infusion at 0.5–0.7 μg · kg^−1^ · h^−1^ is a standard regime for intraoperative use of dexmedetomidine, and is most widely reported in use for dexmedetomidine sedation for AFOI [12, 16–21]. A higher dose may cause hypertension [22], while a lower one may not achieve adequate sedation. The reported target concentrations of remifentanil for AFOI vary [9, 10, 23–30]; in most studies the target effect site concentration of remifentanil at the time of endotracheal intubation was 2–4 ng · ml^−1^ [9, 10, 23–28, 30]. This informed our choice of 3 ng · ml^−1^.

We found that the main differences between the groups were sedation level and ETT tolerance. The patients in Group D were more deeply sedated, but patients in both groups were able to cooperate with the operator during airway anesthesia. There were no significant differences in the first intubation attempt success rates or the overall score for intubation conditions at the first attempt between the groups. After intubation, the proportion of patients who coughed mildly in Group D was twice that of Group R. Although patients in Group D were significantly less likely to tolerate the ETT, there was nonetheless no difference in the proportion with hypertension or tachycardia compared with Group R. The apparent lack of a profound hemodynamic response to coughing and the higher incidence of bradycardia in Group D may be explained by the anti-sympathetic effect of dexmedetomidine.

In contrast, Hu and colleagues found that dexmedetomidine and remifentanil were both effective in patients undergoing awake fiberoptic nasotracheal intubation, and there were no significant differences in intubation or post-intubation scores between those sedated with dexmedetomidine versus remifentanil [9]. Unlike a flexible FOB, the SOS is a more rigid rod with limited degrees of freedom. This difference makes the manipulation of the SOS more stimulating than a FOB. Additionally, the SOS does not have a working channel though which local anesthetic can be administered in a ‘spray-as-you-go’ manner. Although the vocal cords and the airway above the vocal cords can be adequately anesthetized through an LMA MADgic, local anesthetic could not be sprayed into the trachea. The suppression of the cough reflex after intubation relied mainly on the analgesic properties of the sedative.

There have been several reports of the use of dexmedetomidine or remifentanil for AFOI without topical anesthesia. In one case report, a loading dose of 1.0 μg · kg^−1^ dexmedetomidine followed by an infusion of 0.6 μg · kg^−1^ · h^−1^ was used as the sole agent for AFIO in a patient with local anesthetic allergy [18]. The authors reported that the patient tolerated the procedure well with minimal discomfort despite the lack of topical anesthesia. Our findings suggest that for awake SOS intubation a combination of dexmedetomidine with a more potent analgesic drug should be considered to achieve better ETT tolerance. A recent study showed that for AFOI, the use of dexmedetomidine (1 μg · kg^−1^ loading dose followed by an infusion of 0.5 μg · kg^−1^ · h^−1^) plus ketamine (15 mg loading dose and a 20 mg · h^−1^ continuous infusion) provided better hemodynamic stability and sedation than dexmedetomidine alone [31].

The proportion of patients in our study who experienced mild or prolonged coughing after intubation was significantly lower in Group R than Group D. In a previous study, Song and colleagues used ‘no sustained and repetitive coughing with head lift’ as the indicator of suitable sedation conditions for AFIO in patients undergoing cervical spine surgery [28]. Although in our study,all patients in Group R met Song’s criteria, a higher plasma remifentanil concentration might reduce the incidence of mild coughing further. Vennila and colleagues used a target controlled infusion of remifentanil as the sole agent for AFIO without ‘spray-as-you-go’ local anesthesia [29]. The higher mean remifentanil concentration that they adopted (6.3 ng · ml^−1^ at nasal endoscopy, and 8.06 ng · ml^−1^ during tracheal intubation) was shown to be safe. Although in the present study, Group R had a higher incidence of hypoxia, the degree of hypoxia was modest—the lowest SpO_2_ was 85 % in one patient. Hypoxia was successfully addressed within 2 min in all patients who desaturated. The majority of patients who developed desaturation in both groups had a history of smoking or pulmonary disease, or a BMI >30 kg · m^−2^. Caution should be exercised when administering sedation in such patients.

We found that the proportion of patients who recalled airway management was higher in the remifentanil group than the dexmedetomidine group. This finding chimes with those of previous studies, which have reported that dexmedetomidine has a stronger amnesic effect than remifentanil [9, 10]. Although the recall rate was higher in Group R, most patients did not find the experience unpleasant, reflected in broadly comparable patient satisfaction scores. Although no dental injuries were reported in our study, there was a high incidence of sore throat in both groups (47.1 % versus 52.9 %, *P* = 0.628), which may have been a consequence of surgery rather than airway instrumentation. It has previously been reported that the incidence of sore throat was as high as 51–74 % in patients undergoing cervical spine surgery [32].

Our study had some limitations. First, the sample size in each group was 34 and the primary endpoint was first-attempt intubation success rate. These were 79.4 and 85.3 % in each group, meaning that the risk of a statistical type II error was 0.88. Therefore, further larger samples studies will be needed to confirm our results. Second, there was also a relatively high chance that our sample size was inadequate to detect intergroup differences for uncommon adverse events (e.g., lip or teeth injuries, complications associated with oxygen desaturation events, etc.). Finally, our results cannot be extrapolated to settings where target-controlled infusion of remifentanil is not available.

## Conclusions

Dexmedetomidine and remifentanil are effective sedatives for awake intubation using the SOS. Although the first-attempt success rates were similar between the two groups, patients sedated with remifentanil tolerated the ETT better at the expense of an increased risk of mild hypoxia.

## Abbreviations

AFOI, awake fiberoptic endoscope intubation; DBP, diastolic blood pressure; ETT, endotracheal tube; FOB, fiberoptic endoscope; HR, heart rate; RSS, Ramsay Sedation Scale; SBP, systolic blood pressure; SOS, Shikani optical stylet; TCI, target controlled infusion
